# Proliferative Diabetic Retinopathy Disproportionately Impacts Distressed Communities Near a Northeastern Academic Center

**DOI:** 10.1016/j.xops.2025.100872

**Published:** 2025-06-30

**Authors:** Akua A. Frimpong, Thomas L. Chang, June-Marie Weiss, Brittany G. Assanah-Lewis, Ming-Chen Lu, Kristen Harris Nwanyanwu

**Affiliations:** 1 Department of Ophthalmology, University of Vermont Larner College of Medicine, Burlington, Vermont; 2 Department of Ophthalmology and Visual Science, Yale School of Medicine, New Haven, Connecticut; 3 Department of Internal Medicine, Yale School of Medicine, New Haven, Connecticut; 4 Department of Ophthalmology and Visual Sciences, University of Michigan, Ann Arbor, Michigan

**Keywords:** Disease progression, Proliferative diabetic retinopathy, Social deprivation indices, Social determinants of health

## Abstract

**Purpose:**

To identify the associations between social determinants of health (SDoH) and the progression of proliferative diabetic retinopathy (PDR).

**Design:**

Secondary analysis of a retrospective cohort study.

**Participants:**

We extracted data from electronic medical records of individuals at the Yale Eye Center or Dana Eye Clinic, ages ≥18 years, who had a documented diagnosis of nonproliferative diabetic retinopathy (NPDR) at their first recorded (index) ophthalmology visit within the study period.

**Methods:**

We identified participants with NPDR whose disease progressed to PDR during the study time period. We assigned Distressed Communities Index (DCI) scores using participants’ zip codes and created a visualized geographic distribution of scores using ArcGIS. We assessed differences in sociodemographic and health characteristics between participants whose disease progressed to PDR and those whose disease did not progress using 2-sample *t* tests, chi-square, and Fisher exact tests where appropriate. We used logistic regression to assess the associations between SDoH and progression to PDR. We conducted a time-to-event analysis using Cox proportional hazards regression, adjusting for relevant confounders.

**Main Outcome Measures:**

The primary outcome was the progression from NPDR to PDR.

**Results:**

Among the 1354 participants, 137 (10%) developed PDR within the study's 7-year period. Of the 137, 54% were male, 46% were aged ≥65 years, 35% identified as White or Caucasian, and 34% identified as Black or African American. Those whose disease progressed to PDR had significantly worse DCI scores compared to those whose disease did not progress (mean [standard deviation 64 (26) vs. 58 (27), *P* = 0.015). Unadjusted logistic regression revealed a significant association between DCI and progression to PDR (*P* = 0.037), whereas the adjusted model did not (*P* = 0.124).

**Conclusions:**

Participants with disease progression to PDR were more likely to live in disadvantaged areas. Using socioeconomic data and geographic mapping to identify high-risk populations may help health care professionals implement early screening and provide better resources for those at risk of retinal disease progression.

**Financial Disclosure(s):**

Proprietary or commercial disclosure may be found in the Footnotes and Disclosures at the end of this article.

Diabetic retinopathy (DR) is a neurovascular complication of diabetes and the primary cause of severe vision impairment among working-age adults in the Western world. Over 37 million people in the United States are living with diabetes, putting them at serious risk for both macrovascular and microvascular complications.^1^ As of 2021, an estimated 9.6 million Americans have been affected by DR.^2^ It is estimated that, by 2050, the number of individuals with DR in the United States may reach 16 million, and 3.4 million of them will face severe visual complications.^3^ The key to preventing blindness is early detection and timely treatment. The rise in diabetes prevalence presents an urgent need to explore the factors associated with DR and to develop effective early detection and management strategies.^4^^,^^5^

Social determinants of health (SDoH) are the nonmedical factors influencing health outcomes, including features of the environments in which people are born, and where they grow, live, work, and age. These determinants also extend to broader influences and structures that affect daily living conditions, including economic policies, social norms, development agendas, social policies, racism, climate change, and political systems.^6^ Research shows that race, ethnicity, and SDoH significantly impact health outcomes in individuals with diabetes.^7^ The examination of SDoH in relation to DR reveals how socioeconomically disadvantaged individuals are disproportionately affected.^8^^,^^9^

The Distressed Communities Index (DCI) captures various facets of SDoH, including educational attainment and median income, and serves as a vital tool in assessing socioeconomic status.^10^^,^^11^ Geographic Information Systems technology supports the analysis of geographic distributions of health conditions and the identification of at-risk areas and populations, facilitating targeted public health interventions and resource allocation.^12^^,^^13^

## Methods

We conducted a retrospective study of patients with a documented diagnosis of nonproliferative DR (NPDR) who received care at 2 clinics within an urban academic health system in New Haven, CT (Yale Eye Center and Dana Eye Clinic). We extracted data from electronic health records spanning 7 years (January 1, 2013, to April 1, 2020) to assess and refine the predictive capacity of a risk score algorithm developed by Nwanyanwu et al.^14^

We used International Classification of Diseases (ICD) codes, as shown in Table 1 (supplemental, available at www.ophthalmologyscience.org), from initial comprehensive ophthalmic assessments to determine if participants’ disease progressed to proliferative DR (PDR). We included participants in the study if they (1) were ≥18 years old, (2) had ≥2 separate encounters with an ophthalmologist during the study period spaced ≥1 year apart, and (3) had a documented diagnosis of NPDR at their first recorded (index) visit within the study period. We excluded participants who were <18 years at their initial visit or had a pre-existing PDR diagnosis.

**Table 1:** ICD-9 and ICD-10 Codes Corresponding to NPDR and PDR Diagnoses

NPDR	ICD-9: 362.01, 362.03, 362.04, 362.05, 362.06; ICD-10: E08.32XX∗, E08.33XX, E08.34XX, E09.32XX, E09.33XX, E10.34XX, E10.32XX, E10.33XX, E10.34XX, E11.32XX, E11.33XX, E11.34XX, E13.32XX, E13.33XX, E13.34XX
PDR	ICD-9: 362.02; ICD-10: E08.35XX, E09.35XX, E10.35XX, E11.35XX, E13.35X

ICD = International Classification of Diseases; NPDR = nonproliferative diabetic retinopathy; PDR = proliferative diabetic retinopathy.

∗ “XX” represents variable digits in ICD9 and ICD10 codes that are required to make the codes billable.

We collected data on baseline sociodemographic characteristics (age at encounter, current age, sex, ethnicity, race, primary language spoken, primary care physician, insurance coverage, and residential zip code), clinical factors (hemoglobin A1c [HbA1c] levels, diagnoses of uncomplicated and complicated hypertension, dyslipidemia, diabetic neuropathy, diabetic nephropathy, and nonhealing ulcers), and additional variables (days since the first NPDR visit and follow-up PDR status based on ICD9 and ICD10 codes).

### Evaluating Economic Well-Being on a Community-Level Scale: Distressed Communities Index

We utilized ArcGIS 10.6.1 (Environmental Systems Research Institute, Inc.) to associate geographical coordinates with each participant’s zip code. We linked participant’s zip codes to the 2020 Zip Code Tabulation Area TIGER/Line Shapefiles.^15^ We created maps illustrating the geographic distributions of participants diagnosed with NPDR and PDR. Finally, we generated a map to compare disease progression across communities with distinct levels of economic well-being.

Utilizing the Economic Innovation Group's methodology, we assigned a DCI distress score from 0 (most prosperous) to 100 (most distressed). This score reflects seven key factors: average education level, number of housing vacancies, general employment statuses, poverty rates, income ratios, rates of employment changes, and numbers of business establishment changes, offering a comprehensive measure of community economic health. We divided the cohort into quintiles according to the classification provided by the Economic Innovation Group, which designated communities as prosperous (0–20.00), comfortable (20.01–40.00), mid-tier (40.01–60.00), at-risk (60.01–80.00), or distressed (80.01–100) based on their DCI distress scores. We obtained a final dataset incorporating 1343 zip codes. The final sample included a total cohort of participants residing in 170 unique zip codes with DCI distress scores available for 169 unique zip codes.

### Ethical Statement

The Institutional Review Board at Yale School of Medicine approved this study (IRB#2000027927). All research procedures adhered to the ethical principles outlined in the Declaration of Helsinki and institutional guidelines for human research. Given the retrospective nature of this study and the use of deidentified data, the Institutional Review Board waived the requirement for informed consent. All data were fully deidentified before analysis, and the authors ensured data confidentiality in compliance with institutional and regulatory guidelines.

### Statistical Analysis

We summarized participants’ demographic characteristics using descriptive statistics. We used 2-sample *t* tests for continuous measures and chi-square or Fisher exact tests for categorical measures to identify differences between those whose disease progressed to PDR versus those whose disease did not progress. *P* values < 0.05 were considered statistically significant. Significant chi-square or Fisher exact tests were followed by post hoc Holm-adjusted pairwise comparisons. We used logistic regression models to assess the probability of participants progressing to PDR and examine associations between SDoH factors and the likelihood of developing PDR. We reported model estimates as odds ratios (OR) and 95% confidence intervals (CIs). We did not perform a priori sample size calculations because the present work incorporates all available data. We used univariate analysis of variance to compare the mean DCI differences across racial groups among individuals with and without disease progression. We conducted a time-to-event analysis to assess the association between SDOH, measured by DCI quintiles, and progression from NPDR to PDR. We used Cox proportional hazards regression and checked proportionality assumptions. We also conducted a Kaplan–Meier survival analysis, stratifying participants by DCI quintile, to visualize differences in time to progression from NPDR to PDR. We used the log-rank test to compare survival distributions across groups. Additional details are provided in the Supplementary Appendix (Appendix A, available at www.ophthalmologyscience.org; Table 2). We performed these analyses using the IBM Statistical Package for the Social Sciences version 29.0.^16^ We handled missing data using list-wise deletion and assumed it to be missing at random. Several variables in the dataset contained missing data, including HbA1c (14% missing), insurance (5% missing), ethnicity (1% missing), language (<1% missing), race (3% missing), zip code (<1% missing), and DCI distress score (1% missing). To address this, we analyzed only observations with complete data for the variables of interest, excluding cases in which responses were marked as blank, unknown, null, or the participant refused to answer. However, for insurance status, we retained cases with missing data by categorizing them as “unknown” to preserve sample size and assess whether an undefined insurance status showed an association with progression.

**Table 2:** Cox Regression Model for Risk Factors Associated with Progression to PDR

Variable	Hazard Ratio	95% Confidence Interval		*P* Value§
		Lower	Upper	
Age	0.962	0.952	0.971	<0.001¶
Insurance∗				
Medicare	Reference group	-	-	-
Medicaid	1.450	1.071	1.964	0.016∥
Private	1.387	1.010	1.904	0.043∥
Unknown	2.526	1.542	4.140	<0.001¶
Sex	0.950	0.752	1.201	0.668
Language†				
English	Reference group	-	-	-
Spanish	0.870	0.608	1.244	0.445
Other	0.329	0.127	0.855	0.022∥
Race‡				
Black	Reference group	-	-	-
White	1.179	0.863	1.610	0.301
Asian	2.902	1.288	6.539	0.010∥
Other	2.152	1.484	3.121	<0.001¶
Diabetic neuropathy	0.591	0.457	0.765	<0.001¶
Diabetic nephropathy	0.687	0.517	0.912	<0.009¶
Complicated hypertension	1.402	0.986	1.993	0.060
Uncomplicated hypertension	1.667	1.203	2.310	0.002¶
Dyslipidemia	0.607	0.462	0.798	<0.001¶
Nonhealing ulcer	0.697	0.537	0.904	0.007¶
HgbA1c	1.007	0.991	1.022	0.414
DCI				
Quintile 1 (prosperous)	Reference group	-	-	-
Quintile 2 (comfortable)	10.650	5.693	19.924	<0.001¶
Quintile 3 (mid-tier)	3.390	1.730	6.640	<0.001¶
Quintile 4 (at-risk)	5.631	2.962	10.703	<0.001¶
Quintile 5 (distressed)	7.308	3.822	13.976	<0.001¶

DCI = Distress Communities Index.

Medicaid = Project Access, Medicaid New York, Mashantucket Pequot, Medicaid Connecticut.

Private (including Commercial) = Yale Health, Commercial Generic, AETNA Golden, Connecticare, Cigna, Oxford, BCBS, Local 1199seiu Benefit Funds, Meritain Health, Diversified Admin, UMR, GEHA, The Hartford, United Healthcare, Harvard Pilgrim.

∗ Medicare = Tricare, Program Veteran Administration, Wellcare Choice MCR MGD, BCBS MCR MGD, United Healthcare MCR MGD, Medicare, AETNA Golden MCR MGD, Connecticare MCR MGD, Railroad Medicare.

† Other = Arabic, Vietnamese, Turkish, Sign Language, French, Haitian Creole, Korean, Farsi; Persian, Polish, Chinese, Cantonese (inc Toishanese), Greek, Punjabi, Creole, Thai, Hebrew, Philippine (other), Bengali, Portuguese, Albanian, Cambodian, Italian, Laotian, Amharic, Pashto, German, Nepali, Tigrinya, Urdu Pakistan, Cape Verde Creole.

‡ Other = American Indian, Alaska Native, Native Hawaiian, Pacific Islander, other.

§ *P* values were calculated using *t* tests for continuous variables and chi-square tests for categorical variables.

∥ *P* < 0.05.

¶ *P* < 0.01.

## Results

### Participant Characteristics

**Figure 1:**
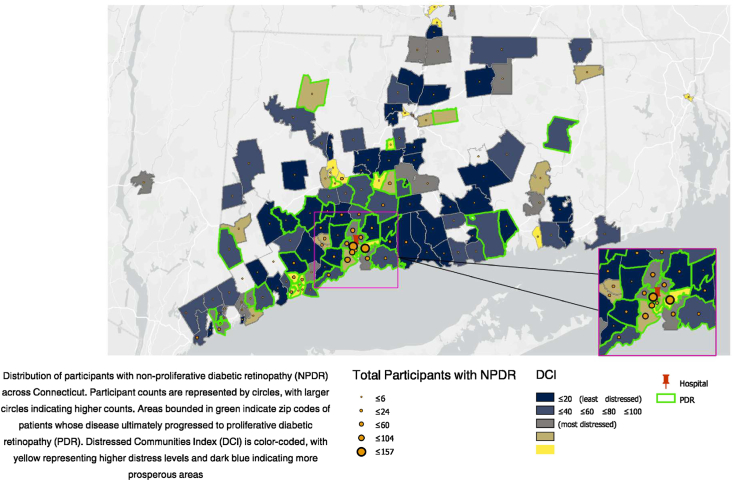
Map of participant zip codes (Connecticut and surrounding areas), indicating both progression to PDR and nonprogression, with corresponding DCI distress scores. This map shows the distribution of participants with NPDR across Connecticut. Participant counts are represented by circles, with larger circles indicating higher counts. Areas bounded in green indicate zip codes of participants whose disease ultimately progressed to PDR. DCI is color-coded, with yellow representing higher distress levels and dark blue indicating more prosperous areas.^17^ DCI = Distressed Communities Index; NPDR = non-proliferative diabetic retinopathy; PDR = proliferative diabetic reintopathy.

We included 1354 eligible participants in this study. Participants had a mean age of 66 years (standard deviation = 13.9), 52% identified as women, 43% identified as White, 33% identified as Black or African American, and 28% identified as Hispanic or Latino. The mean DCI distress score of our sample was 58 (26.9). Over half of the participants lived in areas with high DCI distress scores, (i.e., more distressed areas), 20% lived in areas with moderate DCI distress scores, and conversely, around 25% came from zip codes with lower DCI distress scores. Figure 1 maps participants' residence, the socioeconomic distress levels of each area, and the regions where participants with disease progression resided, using a map created in ArcGIS 10.6.1.^17^

**Figure 2:**
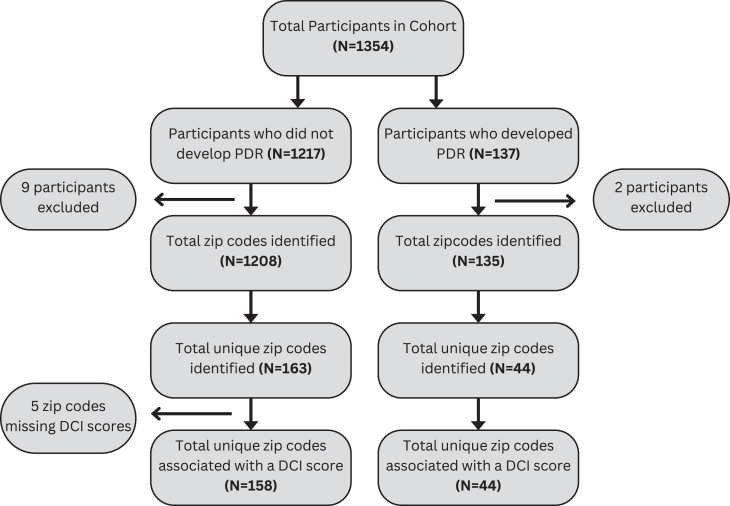
Cohort flow chart depicting the numbers of participants included in the analysis and the number of unique Distressed Communities Index distress scores. DCI = Distressed Communities Index; PDR = proliferative diabetic retinopathy.

Among the cohort, approximately 10% (n = 137), mean age at encounter = 58 [13] developed PDR during their follow-up visits over the study timeframe. Of the participants with PDR, 46% (n = 63) were women, 37% (n = 48) White, 35% (n = 46) Black, 4% (n = 5) Asian, and 35% (n = 48) of the participants identified as Hispanic or Latino. Participants' sociodemographic and clinical characteristics are summarized and stratified by progression to PDR, are summarized in Table 3. Among the participants whose disease progressed to PDR, zip codes were available for 135 individuals (99%). Figure 2 depicts the zip codes of participants whose disease progressed to PDR, alongside the DCI distress scores associated with those zip codes.

### Associations with Progression to PDR

Chi-square tests revealed significant associations between the likelihood of developing PDR and several factors: a history of complicated hypertension (*P* = 0.014), characterized by hypertension with one or more complications causing organ damage (such as to the heart, kidneys, brain, and eyes), insurance coverage (*P* < 0.001), ethnicity (*P* = 0.032). Using *t* tests revealed significant associations between the likelihood of developing PDR and several factors, including DCI distress score (0.015) and the age at encounter (*P* = 0.005). Sex, race, language spoken, insurance status, HbA1c, and whether the participant had uncomplicated hypertension, dyslipidemia, diabetic nephropathy, diabetic neuropathy, a nonhealing ulcer, or access to a primary care physician were not significantly associated with disease progression to PDR (see Table 3).

**Table 3:** Descriptive Analysis and Demographics Stratified by PDR Development

Baseline Characteristics	Overall (n = 1354)	No PDR (n = 1217)	PDR (n = 137)	*P* Value§
Age (years), mean (SD)	66 (13.9)	62 (14.0)	58 (13.0)	0.005¶
DCI distress score, mean (SD)	58 (26.9)	58 (26.9)	64 (26.0)	0.015∥
HbA1c (%), mean (SD)	9 (5.8)	9 (6.1)	10 (2.4)	0.291
Sex, n (%)				0.164
Female	699 (51.6)	636 (52.3)	63 (46.0)	
Male	655 (48.4)	581 (47.7)	74 (54.0)	
Race∗, n (%)				0.48
White	563 (43.1)	515 (43.8)	48 (36.9)	
Black or African American	432 (33.1)	386 (32.8)	46 (35.4)	
Other or not listed	264 (20.2)	233 (19.8)	31 (23.8)	
Asian	48 (3.7)	43 (3.7)	5 (3.8)	
Ethnicity, n (%)				0.032∥
Non-Hispanic	969 (72.5)	881 (73.4)	88 (64.7)	
Hispanic or Latino/a/x	368 (27.5)	320 (26.6)	48 (35.3)	
Language†, n (%)				0.135
English	1040 (76.8)	943 (77.5)	97 (70.8)	
Spanish	257 (19.0)	222 (18.2)	35 (25.5)	
Other	57 (4.2)	52 (4.3)	5 (3.6)	
Insurance‡, n (%)				<0.001¶
Medicare	555 (41.0)	518 (42.6)	37 (27.0)	
Medicaid	440 (32.5)	384 (31.6)	56 (40.9)	
Private	285 (21.0)	257 (21.1)	28 (20.4)	
Unknown	74 (5.5)	58 (4.8)	16 (11.7)	
DCI, n (%)				0.037∥
0–20 (prosperous)	173 (12.9)	163 (13.5)	10 (7.4)	
21–40 (comfortable)	169 (12.6)	151 (12.6)	18 (13.3)	
41–60 (mid-tier)	275 (20.6)	254 (21.1)	21 (15.6)	
61–80 (at-risk)	346 (25.9)	310 (25.8)	36 (26.7)	
81–100 (distressed)	375 (28.0)	325 (27.0)	50 (37.0)	
Primary care physician, n (%)				0.144
Yes	1304 (96.3)	1169 (96.1)	135 (98.5)	
No	50 (3.7)	48 (3.9)	2 (1.5)	
Uncomplicated hypertension, n (%)				0.275
Yes	811 (59.9)	723 (59.4)	88 (64.2)	
No	543 (40.1)	494 (40.6)	49 (35.8)	
Complicated hypertension, n (%)				0.014∥
Yes	69 (5.1)	56 (4.6)	13 (9.5)	
No	1285 (94.9)	1161 (95.4)	124 (90.5)	
Dyslipidemia, n (%)				0.608
Yes	621 (45.9)	561 (46.1)	60 (43.8)	
No	733 (54.1)	656 (53.9)	77 (56.2)	
Diabetic nephropathy, n (%)				0.097
Yes	246 (18.2)	214 (17.6)	32 (23.4)	
No	1108 (81.8)	1003 (82.4)	105 (76.6)	
Diabetic neuropathy, n (%)				0.784
Yes	372 (27.5)	333 (27.4)	39 (28.5)	
No	982 (72.5)	884 (72.6)	98 (71.5)	
Nonhealing ulcer, n (%)				0.333
Yes	283 (20.9)	250 (20.5)	33 (24.1)	
No	1071 (79.1)	967 (79.5)	104 (75.9)	

DCI = Distress Communities Index; HbA1c = hemoglobin A1c; PDR = proliferative DR; SD = standard deviation.

Medicaid = Project Access, Medicaid New York, Mashantucket Pequot, Medicaid Connecticut.

Private (including Commercial) = Yale Health, Commercial Generic, AETNA Golden, Connecticare, Cigna, Oxford, Blue Cross Blue Shield, Local 1199seiu Benefit Funds, Meritain Health, Diversified Admin a UnitedHealthcare company, Government Employees Health Association, The Hartford, United Healthcare, Harvard Pilgrim.

∗ Other = American Indian, Alaska Native, Native Hawaiian, Pacific Islander, other.

† Other = Arabic, Vietnamese, Turkish, Sign Language, French, Haitian Creole, Korean, Farsi; Persian, Polish, Chinese, Cantonese (inc Taishanese), Greek, Punjabi, Creole, Thai, Hebrew, Philippine (other), Bengali, Portuguese, Albanian, Cambodian, Italian, Laotian, Amharic, Pashto, German, Nepali, Tigrinya, Urdu Pakistan, Cape Verde Creole.

‡ Medicare = Tricare, Program Veteran Administration, Wellcare Choice Medicare Managed Care (MCR MGD), BCBS MCR MGD, United Healthcare MCR MGD, Medicare, AETNA Golden MCR MGD, Connecticare MCR MGD, Railroad Medicare.

§ *P* values were calculated using *t* tests for continuous variables and chi-square tests for categorical variables.

∥ *P* < 0.05.

¶ *P* < 0.01.

The unadjusted logistic regression analysis (Table 4) revealed a significant association between DCI quintiles and progression to PDR (*P* = 0.037). Individuals in quintile 5 (most distressed) had a 2.5 times higher odds of progressing to PDR (OR, 2.51; 95% CI, 1.24–5.07; *P* = 0.011) compared to those in quintile 1 (most prosperous). Table 5 presents the adjusted logistic regression analysis examining the association of various factors with PDR. Initial correlation analysis revealed an association between the DCI and several demographic and clinical variables, including uncomplicated hypertension, nonhealing ulcers, primary language, and insurance. After adjusting for covariates, including age at the encounter, insurance, sex, language, race, diabetic nephropathy, diabetic neuropathy, hypertension (uncomplicated and complicated), dyslipidemia, nonhealing ulcers, and HbA1c, the overall association between DCI and disease progression to PDR was no longer statistically significant (*P* = 0.124). Participants in quintile 2 (comfortable) (OR, 2.76; 95% CI 1.03–7.43; *P* = 0.044) and quintile 5 (most distressed) (OR, 2.84; 95% CI, 1.09–7.36; *P* = 0.032) had significantly higher odds of progressing to PDR. Those with complicated hypertension were more than twice as likely to develop PDR (OR, 2.11; 95% CI, 1.06–4.20; *P* = 0.033), as well as those with uncomplicated hypertension faced an increased risk (OR, 1.72; 95% CI, 1.01–2.93; *P* = 0.045). Participants with unknown insurance had the highest risk of progression (OR, 4.06; 95% CI, 1.73–9.48; *P* = 0.001) in comparison to those with Medicare.

**Table 4:** Unadjusted Logistic Regression Analysis for Factors Associated with Progression to Proliferative DR

Variable	Odds Ratio	95% Confidence Interval		*P* Value∗
		Lower	Upper	
DCI				
Quintile 1 (prosperous)	Reference group	—	—	—
Quintile 2 (comfortable)	1.943	0.869	4.342	0.105
Quintile 3 (mid-tier)	1.348	0.619	2.935	0.452
Quintile 4 (at-risk)	1.893	0.916	3.912	0.085
Quintile 5 (distressed)	2.508	1.240	5.073	0.011†

DCI = Distress Communities Index.

*P* < 0.01 compared with Quintile 1 (reference group).

∗ *P* values were calculated using logistic regression.

† *P* < 0.05.

**Table 5:** Adjusted Logistic Regression Analysis for Factors Associated with Progression to Proliferative DR

Variable	Odds Ratio	95% Confidence Interval		*P* Value§
		Lower	Upper	
Male	1.122	0.742	1.696	0.587
Age	0.990	0.972	1.008	0.271
Insurance∗				
Medicare	Reference group	-	-	-
Medicaid	1.722	0.999	2.970	0.051
Private (commercial)	1.481	0.783	2.803	0.227
Unknown	4.055	1.734	9.481	0.001¶
Language†				
English	Reference group	-	-	-
Spanish	1.133	0.609	2.109	0.692
Others	0.496	0.127	1.943	0.314
Race‡				
Black	Reference group	-	-	-
White	1.010	0.593	1.720	0.971
Asian	1.207	0.345	4.222	0.768
Others	1.061	0.547	2.057	0.861
Diabetic neuropathy	0.837	0.516	1.358	0.472
Diabetic nephropathy	1.420	0.866	2.330	0.165
Nonhealing ulcer	1.266	0.777	2.064	0.344
Dyslipidemia	0.750	0.472	1.191	0.223
Complicated hypertension	2.113	1.064	4.195	0.033∥
Uncomplicated hypertension	1.723	1.013	2.931	0.045∥
HbA1c	1.010	0.986	1.034	0.433
DCI				
Quintile 1 (prosperous)	Reference group	-	-	-
Quintile 2 (comfortable)	2.764	1.028	7.431	0.044∥
Quintile 3 (mid-tier)	1.616	0.596	4.385	0.346
Quintile 4 (at-risk)	2.363	0.908	6.151	0.078
Quintile 5 (distressed)	2.836	1.092	7.361	0.032∥

DCI = Distress Communities Index.

Medicaid = Project Access, Medicaid New York, Mashantucket Pequot, Medicaid Connecticut.

Private (including Commercial) = Yale Health, Commercial Generic, AETNA Golden, Connecticare, Cigna, Oxford, BCBS, Local 1199seiu Benefit Funds, Meritain Health, Diversified Admin, UMR, GEHA, The Hartford, United Healthcare, Harvard Pilgrim.

∗ Medicare = Tricare, Program Veteran Administration, Wellcare Choice MCR MGD, BCBS MCR MGD, United Healthcare MCR MGD, Medicare, AETNA Golden MCR MGD, Connecticare MCR MGD, Railroad Medicare.

† Other = Arabic, Vietnamese, Turkish, Sign Language, French, Haitian Creole, Korean, Farsi; Persian, Polish, Chinese, Cantonese (inc Toishanese), Greek, Punjabi, Creole, Thai, Hebrew, Philippine (other), Bengali, Portuguese, Albanian, Cambodian, Italian, Laotian, Amharic, Pashto, German, Nepali, Tigrinya, Urdu Pakistan, Cape Verde Creole, Unknown.

‡ Other = American Indian, Alaska Native, Native Hawaiian, Pacific Islander, Other.

§ *P* values were calculated using logistic regression.

∥ *P* < 0.05.

¶ *P* < 0.01.

Results from analysis of variance found statistically significant differences in DCI distress scores between racial groups (*P* < 0.001). The results of a Tukey post hoc test showed that distinct patterns emerged when comparing DCI distress scores between racial groups. Participants who identified as Black exhibited higher DCI distress scores than those who self-identified as White (*P* < 0.001), Asian (*P* < 0.001), or other (uncategorized) (*P* < 0.001). Moreover, participants identifying as Black or other exhibited significantly higher DCI distress scores compared to those identifying as Asian (82.579 (3), respectively, all *P* < 0.001), as shown in Table 6.

**Table 6:** Average Differences in DCI Distress Scores by Race

Race	Comparison Race	Mean Difference	Standard Error	*P* Value∗	95% Confidence Interval	
					Lower	Upper
Black	White	20.655	1.586	<0.001‡	16.5752	24.7348
	Asian	20.188	3.7499	<0.001‡	10.542	29.8342
	Other	−2.4857	1.9324	0.571	−7.4567	2.4853
White	Black	−20.655	1.586	<0.001‡	−24.7349	−16.5752
	Asian	−0.4669	3.7069	0.999	−10.0025	9.0686
	Other	−23.1407	1.8476	<0.001‡	−27.8936	−18.3879
Asian	Black	−20.1881	3.7499	<0.001‡	−29.8342	−10.542
	White	0.4669	3.7069	0.999	−9.0686	10.002
	Other	−22.6738	3.8631	<0.001‡	−32.6233	−12.7243
Other†	Black	2.4857	1.9324	0.572	−2.4853	7.4567
	White	23.1407	1.8476	<0.001‡	18.3879	27.8936
	Asian	22.6738	3.8678	<0.001‡	12.7243	32.6233

DCI = Distress Communities Index.

∗ *P* values were calculated using a 1-way analysis of variance, followed by a Tukey post hoc test.

† Other = American Indian, Alaska Native, Native Hawaiian, Pacific Islander, other.

‡ *P* < 0.001.

## Discussion

This study found that participants whose disease progressed to PDR exhibited differences in age, pre-existing hypertension, insurance coverage, and ethnicity. Participants residing in neighborhoods with higher distress scores were more than twice as likely to develop PDR than those in neighborhoods with low distress scores. Participants identifying as Black or African American or categorized under other or not listed race displayed the highest mean DCI distress scores. The association between distress scores and disease progression emphasizes the value of examining community-level factors in understanding clinical outcomes.

Understanding the impact of SDoH on the progression of DR is crucial because effective management requires more than just the knowledge of the pathophysiology of the disease but also involves recognizing the broader environmental and societal factors influencing patient outcomes. Identifying geographic regions with elevated risk of PDR allows for precise interventions focused on bolstering resource accessibility, augmenting the prevalence of eye screenings, and enhancing diabetes management and diabetic eye care services. One study utilized Geographic Information System mapping to visualize the geographic distribution of primary care physicians and ophthalmologists across North Carolina and found that geographic and road network factors impact patient accessibility to these physicians.^18^ Other studies utilized Geographic Information System in ophthalmology to visualize and address access barriers to eye care for patients with diabetes, further emphasizing its utility in improving patient outcomes.

Although other works have examined the relationship between PDR and DCI distress scores,^19^ the present study explores granular PDR prevalence using a comprehensive cohort study design. Our results showed that individuals living in socioeconomically distressed areas had twice the odds of developing PDR compared to those residing in more prosperous neighborhoods. Research shows that an individual's environment or neighborhood influences the prevalence of DR.^20^^,^^21^ Factors like race, ethnicity, income, educational attainment, and insurance coverage are known to influence the prevalence of diabetes.^22^, ^23^, ^24^, ^25^ Thorough investigations demonstrate that specific SDoH, like race and ethnicity,^26^, ^27^, ^28^, ^29^ education, and income level^26^^,^^30^ are associated with an increased risk of DR.

Our study builds upon existing findings by employing an analytical approach that integrates an index specifically designed to assess the impacts of certain SDoH on the progression to PDR. Using DCI distress scores to study relationships between disease progression and SDoH is a novel technique. It allows researchers to gain new insights into how to prevent blindness from DR. Our focus on those with NPDR provides insights into unique challenges impacting the worsening of DR.

### Sociodemographic and Clinical Characteristics

Our analysis revealed key demographic and clinical factors associated with the progression of NPDR to PDR. We observed that participants whose disease progressed to PDR were more often male, had a higher HbA1c, and tended to be slightly younger than those whose disease did not progress to PDR. We also noted that factors like insurance coverage, ethnicity, and a history of complicated hypertension influenced progression to PDR. Previous studies have shown that these factors contribute to the progression of DR and increase the risk of the disease.^31^^,^^32^

Although the unadjusted model showed that individuals living in more distressed communities (higher DCI quintiles) were more likely to progress to PDR, we found that this association was no longer statistically significant after adjusting for demographic and clinical variables in our dataset. This suggests that the effect of community-level socioeconomic distress may be mediated through or confounded by additional factors. The DCI in this model captured overlapping information with several covariates, such as insurance status, language, and comorbid conditions, because it functions as a proxy for individual-level socioeconomic or health-related disadvantages. Rather than being a direct driver of disease progression, DCI may reflect a broader context of social deprivation that indirectly influences health outcomes by increasing the prevalence of comorbidities associated with PDR. The worsening of diabetes-related outcomes in these communities is likely rooted in the systemic impact of social disadvantage.^33^ Living in a distressed community may be linked to reduced access to care, poorer overall health status, and other structural barriers that cumulatively impact diabetes management and complications. These findings also highlight the importance of considering both individual and community-level factors when assessing risk and designing interventions for DR.

Prior research has demonstrated that individuals living in high-distress communities are more likely to experience multiple barriers to optimal diabetes management, including reduced health care access, delayed screening, poor glycemic control, food insecurity, and increased burden of chronic diseases—all of which are major risk factors for disease progression.^34^

### Race

Race was not significantly associated with disease progression; however, participants who identified as Black or African American or who were categorized under “other/not listed” were more likely to live in distressed areas. We posit that the large proportion of participants labeled as other/not listed race could be attributed to the inclusion of individuals with multiple races or those who identify as multiracial, a category not explicitly represented in our dataset.^35^ These areas tended to exhibit several SDoH associated with disease progression.^36^

According to the Centers of Disease Control and Prevention National Diabetes Statistics Report from 2019 to 2021, among the US adults aged ≥18, the highest prevalence of diagnosed diabetes was observed in American Indian and Alaska Native adults (13.6%), followed by non-Hispanic Black adults (12.1%), Hispanic adults (11.7%), non-Hispanic Asian adults (9.1%), and Non-Hispanic White adults (6.9%).^37^ Among the 1.84 million individuals living with vision-threatening DR, Black and Hispanic individuals exhibit a higher standardized prevalence of vision-threatening DR, with rates of 8.66% and 7.14%, respectively, compared to White individuals, whose prevalence is 3.55%.^2^ Other studies demonstrated that minoritized groups, especially Black people, are at a substantially increased risk of developing diabetes and its associated microvascular complications, like nephropathy and retinopathy.^26^^,^^33^ The Third National Health and Nutrition Examination Survey study also found that DR prevalence and HbA1c values to be significantly greater in non-Hispanic Blacks and Hispanics compared to Non-Hispanic Whites.^38^^,^^39^ Other studies highlighted racial disparities in diabetes,^40^ diabetic macular edema,^41^ and PDR, with a higher prevalence^42^ and increased risk noted in Black patients compared to White patients.^33^ These conditions, shaped by racial disparities, restrict health care access and highlight socioeconomic factors as critical drivers of the disparities in DR rates among racial and ethnic groups.^32^

The increased presence of Black individuals in distressed areas reflects systemic racism. Health disparities among minoritized groups are primarily due to varied exposures to behavioral, psychosocial, environmental, and material risks. Interdisciplinary studies suggest that race has limited direct biological relevance to disease progression, indicating that genetic or racial factors are not inherent determinants of health outcomes.^43^ These studies have highlighted that human racial classification is more a societal perception than a scientific reality.^44^ The Economic Innovation Group 2020 DCI report, which analyzed community well-being from 2000 to 2018, revealed that although some regions and demographic groups saw meaningful progress since the turn of the century, more than half of the 50.5 million Americans living in distressed communities are people of color, emphasizing that the disparities between Black and White households have not only persisted but widened.^45^

Addressing health disparities necessitates understanding SDoH and the impacts of racism to implement public health interventions and societal reforms that mitigate disease risk factors and enhance health equity across racial and ethnic groups. Effective strategies to address these disparities include comprehensive data collection to identify specific contributing factors and implement structural and institutional changes that influence the distribution of disease risk factors.

### Insurance

Compared to patients insured through Medicare, those receiving Medicaid or whose insurance statuses were not reported were significantly more likely to develop PDR. Insurance instability has been linked to worse health outcomes, including with DR.^46^ Future studies could further explore the relationship between insurance status alongside other SDoH to assess the potential impact of policy interventions aimed at improving access to ophthalmologic care.

### Zip Code

Our study demonstrated the utility of 5-digit zip code DCI data. Understanding the relationships between broad geographic areas, SDoH, and disease progression is crucial for future, more detailed neighborhood-level research. Utilizing 5-digit zip codes in our study provided practical benefits, allowing for easy access to and management of large datasets while offering a broad perspective on socioeconomic and demographic trends across wider regions. Furthermore, employing 5-digit zip codes enhances privacy by ensuring anonymity. The geographic distribution in our dataset showcases a variety of community environments, ranging from lower to moderate and significantly distressed areas. This diversity offers valuable insights into the different socioeconomic contexts that influence our participants' health management and disease progression.

### Age

Diabetic retinopathy affects individuals variably across different ages, with severity often escalating due to prolonged diabetes duration and age-related ocular changes. Our findings indicated that participants whose condition progressed to PDR were, on average, 2 years younger than those who did not experience such progression. Nwanyanwu et al^14^ found that individuals with NPDR who progressed to PDR tended to be slightly but significantly younger compared to those who did not progress. Prior research has shown that factors such as younger age at the diagnosis of diabetes and longer disease duration are associated with an increased risk of retinopathy.^47^ The slight age difference observed may only hold clinical significance after considering these factors. Further research is needed to determine the clinical implications of age differences in disease management and outcomes for DR.

### Targeted Interventions

Integrating targeted high-risk interventions with broader public health strategies can enhance the efficiency of limited healthcare resources in diabetes prevention. Evidence strongly supports the cost-effectiveness of primary prevention through intensive lifestyle modifications, as demonstrated by several well-conducted trials, including the United States Diabetes Prevention Program,^48^ Finnish Diabetes Prevention Study,^49^ China Da Qing Diabetes Prevention Study,^50^ and Indian Diabetes Prevention Program.^51^ Zhou et al conducted a systematic review of cost effectiveness interventions to prevent diabetes and found that in-person lifestyle interventions (e.g., group interventions, population-based strategies, and targeted screenings) are associated with some of the strongest cost effectiveness outcomes. By focusing on individuals at the highest risk while simultaneously implementing population-wide measures, this approach ensures a more effective and sustainable impact on reducing the burden of diabetes.^52^

### Study Limitations

Although this study has several strengths, there are some limitations to our approach. First, the study's retrospective design cannot establish a causal relationship between SDoH and PDR. Second, individual-level SDoH was not available for the present sample. Instead, we used area-level data to assess community SDoH, aligning with current literature.^53^, ^54^, ^55^ Although individual-level SDoH data have been shown to improve strengths of association of various outcomes, area-level data can develop indicators for community health and quality of life, offering insights to enhance population health regionally or nationally.^56^^,^^57^

Third, this study may be subject to selection bias due to the inclusion and exclusion criteria. Specifically, individuals with NPDR may have been undercounted because we included only those diagnosed at their initial visit, potentially excluding those diagnosed later. International Classification of Diseases codes have traditionally been used to identify patients with NPDR; many studies rely on structured data including ICD diagnosis codes for case identification.^58^ Reliance on ICD codes for identifying NPDR may introduce misclassification (e.g., human error, broad categories like “unspecified”)^59^ or missed eligible participants. Multiple studies have used ICD9 and ICD10 codes to define DR.^60^ A study concluded that the transition from ICD9 to ICD10 codes was particularly more accurate in distinguishing between NPDR and PDR.^61^ Another recent study demonstrated high concordance between ICD10 codes and documented chart standards of DR.^61^ Future studies could explore the inclusion of participants identified through alternative or additional coding systems to ensure broader representation and further validate the results.

Although the DCI appeared to have some effect, a larger dataset would be necessary to determine whether this association remains consistently observable across populations. In this study, we used DCI as a proxy. This approach may be especially useful in datasets that lack detailed clinical or demographic information, offering a simplified way to flag patients who may need closer monitoring or additional support.

However, using the DCI and the other variables included in our model did not fully explain the likelihood of progression to PDR, suggesting that other unmeasured or more specific factors may contribute to disease progression. It is also possible that additional community-level or individual-level stressors prevalent in distressed neighborhoods were not captured in our dataset. Future research should explore what underlying elements contribute to community distress and how they may influence clinical outcomes in DR.

Finally, using 5-digit codes limits the granularity with which SDoH can be assessed compared to their 9-digit counterparts. Access to a finer neighborhood resolution may allow researchers to identify regions marked by concentrated poverty. However, using ZIP Code Tabulation Areas offers practical geographic information and a sufficiently detailed level of geographic resolution in diverse economic landscapes to assess SDoH while still capturing significant area-level differences.^62^

## Conclusion

Our findings emphasize the necessity of holistic approaches to DR management that consider broader socioeconomic context. Addressing socioeconomic determinants of health through targeted interventions and policy measures may be vital in lowering the risk of progression to PDR in distressed communities.
