# Supplementary material for: Proliferative Diabetic Retinopathy Disproportionately Impacts Distressed Communities Near a Northeastern Academic Center
Source: Ophthalmol Sci. 2025 Jun 30;5(6):100872. doi: 10.1016/j.xops.2025.100872 (PMC12395159; doi:10.1016/j.xops.2025.100872)
Supplement: Appendix A [file mmc1.pdf]

**Appendix A. Additional Data and Details on Time to Event Analysis**

**Results**

*Time to event analysis*

We completed a cox regression model controlling for age at the encounter, insurance, sex, language, race, diabetic nephropathy, diabetic neuropathy, complicated hypertension, (uncomplicated and dyslipidemia, non-healing ulcers, and HbA1c. Compared to Quintile 1 as the reference group, Quintile 2 (HR 10.650, 95% CI 5.69–19.92, $p<0.001$) and Quintile 5 (HR 7.308, 95% CI 3.82–13.98, $p<0.001$) demonstrated a significantly higher hazard. Quintile 3 (HR 3.390, 95% CI 1.73–6.64, $p<0.001$) and Quintile 4 (HR 7.31, 95% CI 3.82–13.98, $p<0.001$) also showed significantly increased hazards. The analysis also revealed that age, ~~race,~~ those with uncomplicated hypertension, diabetic neuropathy, dyslipidemia, and nonhealing ulcer were significantly associated with an increased hazard. Participants insured with Medicaid, Private, or unknown of insurance type were also significantly associated with an increased hazard. No significant association was observed for sex, HbA1c, language, complicated hypertension, language, and diabetic nephropathy (see Table 6)

*Kaplan-Meier Survival Analysis*

The Kaplan-Meier survival analysis demonstrated significant differences in the time to progression to PDR across DCI quintiles (Log-rank test, $p < 0.001$). The survival curves revealed an unexpected crossover pattern, where Quintile 2 had a steeper decline compared to both more and less distressed communities (see Figure 3). Initially, Quintile 5 (Distressed) progressed faster but then Quintile 2 (Comfortable) picks up and crosses over around 167 months. Patients in Quintile 2 exhibited the fastest progression, with a median time to PDR progression of about (188 months, 95% CI: 1936.7–2583.2), followed closely by Quintile 5 (221 months, 95% CI: 2387.5–2916.6). For Quintile 3 and 4, the median survival time was not reached during the study period. In contrast, patients in Quintile 1 had the longest time to

progression, with a median time of 234 months (95% CI: 2540.0–3082.0) and a big cutoff around 217 months.

## Discussion

### *Time to event analysis of Progression to PDR*

In the Kaplan-Meier and multivariable Cox proportional hazards analysis examining the association between the DCI quintiles and progression to PDR, we observed a graded association in which individuals in DCI Quintiles 2 through 5 demonstrated significantly higher hazards of progression compared to those in Quintile 1. Patients residing in Quintile 2 exhibited the highest hazard ratio.

This finding persisted despite adjustment for clinical and demographic variables, including insurance status, HbA1c levels, comorbidities, and visit frequency. The clinical and demographic profiles of patients in Quintile 2 closely resembled those in Quintile 1, suggesting that the observed discrepancy is unlikely to be fully explained by measured covariates.

Given the observational nature of the study, this finding may reflect residual confounding due to unmeasured factors, such as neighborhood-level healthcare fragmentation, environmental exposures, or variations in local healthcare delivery. It is also possible that Quintile 2 represents a distinct subgroup with risk factors not adequately captured in our data. These results underscore the limitations of multivariable adjustment in observational analyses and the potential impact of unmeasured or imprecisely measured confounders on hazard ratio estimates.

The study also highlights the complex and potentially nonlinear relationship between community-level distress and clinical disease progression. Future research is warranted to

explore underlying mechanisms, including qualitative assessments of healthcare access and care continuity, particularly among communities identified as moderately distressed.

## Figure

Figure 3: Kaplan-Meier Survival Curve for Progression to PDR by Distressed Community Index (DCI) Quintiles.

Legend for Figure 3: This Kaplan-Meier survival curve displays the cumulative survival probability for PDR progression, stratified by Distressed Community Index (DCI) quintiles. Time since the initial NPDR visit is used to track disease progression. Each curve represents a different DCI quintile, with lower quintiles (more prosperous areas) and higher quintiles (more distressed communities) compared over time. Censoring events indicate participants lost to follow-up or those who did not progress within the study period.
